# Association of different cell types and inflammation in early acne vulgaris

**DOI:** 10.3389/fimmu.2024.1275269

**Published:** 2024-01-31

**Authors:** Lei Huang, Shuyun Yang, Xiuqin Yu, Fumin Fang, Liping Zhu, Lu Wang, Xiaoping Zhang, Changzhi Yang, Qihong Qian, Tingting Zhu

**Affiliations:** ^1^ Department of Dermatology, The First Affiliated Hospital of Soochow University, Suzhou, China; ^2^ Department of Dermatology, The People’s Hospital of Baoshan, Baoshan, Yunnan, China

**Keywords:** acne vulgaris, inflammation, immune system, *Cutibacterium acnes*, cytokines

## Abstract

Acne vulgaris, one of the most common skin diseases, is a chronic cutaneous inflammation of the upper pilosebaceous unit (PSU) with complex pathogenesis. Inflammation plays a central role in the pathogenesis of acne vulgaris. During the inflammatory process, the innate and adaptive immune systems are coordinately activated to induce immune responses. Understanding the infiltration and cytokine secretion of differential cells in acne lesions, especially in the early stages of inflammation, will provide an insight into the pathogenesis of acne. The purpose of this review is to synthesize the association of different cell types with inflammation in early acne vulgaris and provide a comprehensive understanding of skin inflammation and immune responses.

## 1 Introduction

Acne vulgaris is a common inflammatory dermatosis, affecting approximately 650 million people worldwide (1, 2). Acne can negatively impact the quality of life of patients because of physical and psychosocial morbidities (3). Microcomedones and comedones are primary acne lesions that result from cystic formation in the infundibulum of the pilosebaceous unit (PSU) (4), and the majority of inflammatory lesions arise from comedones, including papule, pustule, nodule and cyst (5). The progression of acne vulgaris may not always occur in a linear manner from microcomedone to inflammatory lesions (6, 7). The etiology of acne is multifactorial and complex, mainly including hyperseborrhea and altered sebum composition, follicular hyperkeratinization, abnormalities of the microbial flora, inflammation and immune responses (8). These factors together can impair the PSU, leading to transformation of normal follicular canals into microcomedones and further progression into inflammatory lesions (9). It is now accepted that inflammation sets in early in the pathogenesis of acne (10).

*Cutibacterium acnes* (*C. acnes*; formerly known as *Propionibacterium acnes*) is a commensal microorganism that resides mainly in the anaerobic portions of the pilosebaceous follicles (11). Although *C. acnes* is observed in normal and acne skin, intense colonization likely causes inflammatory reactions and immune cell recruitment through dysbiosis of the skin microbiome and an imbalance of different *C. acnes* phylotypes (11–13). Based on the sequences of the recA and tly genes, *C. acnes* can be subdivided into phylotypes IA, IB, II and III (14, 15). Multilocus sequence typing (MLST) approaches further divide the type I strain into IA1, IA2, IB and IC clusters, some of which are acne-associated (IA1 and IC) (16, 17). Within microcomedones, which are usually barely visible clinically, *C. acnes* multiplies in the infra-infundibulum, resulting in bacterial colonization (18). *C. acnes* produces many enzymes and biologically active molecules to stimulate immune cells to secrete proinflammatory cytokines. The immune response to *C. acnes*, but not the bacteria itself, has a key role in the pathogenesis of acne (19).

The immune surveillance of the skin barrier is complex. Immune cells account for 7% of the cells in skin under normal conditions (20) and are involved in perceiving alarm signals and orchestrating immune responses when inflammation occurs. Because of the absence of the stratum corneum, the skin appendages become the points of entry for external pathogens, and skin commensal microbiota can extend within the dermis, establishing direct communication with the host immune system (21). The PSU is classified as a site of immune cell recruitment because alteration in microenvironments can impact skin immunobiology (22, 23). The anaerobic and lipophilic microenvironments of the PSU favor the growth of *C. acnes*, particularly in acne vulgaris.

## 2 Inflammation in early acne vulgaris

**Figure 1:**
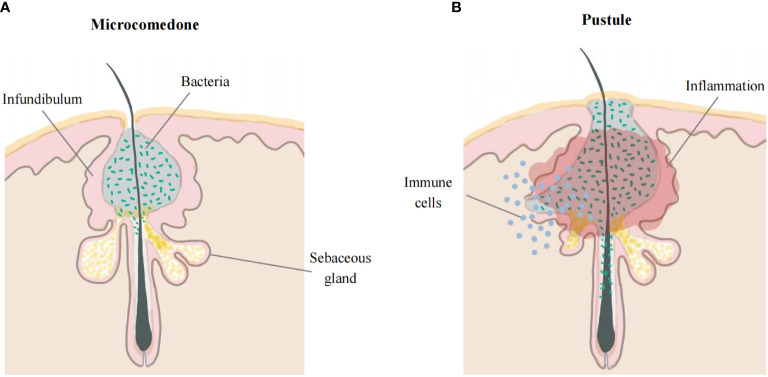
Early acne lesions and late acne lesions. **(A)** The early stage of acne occurs in the hair follicle infundibulum. Microcomedone is mostly composed of lipids with clusters of bacteria, and the outer shell is made up of corneocyte layers. **(B)** The walls of the follicles rupture, leading to extrusion of the content and causing a rapid inflammatory response.

The early stage of acne is characterized by the subclinical microcomedones (5). The interior of microcomedones is mostly composed of lipids with clusters of bacteria, and their outer shell is made up of corneocyte layers (18). Due to increasing pressure from the expansion of the keratin layer in a confined space, hypoxia may facilitate the multiplication of *C. acnes* and lipid accumulation (24, 25). Increased sebum production supports *C. acnes* growth in the PSU. Moreover, the metabolites of bacteria can alter the sebum composition, which contributes to the inflammatory response (26). Eventually, the rupture of the follicular walls causes extrusion of the content and a rapid inflammatory response. Although both CD4^+^ T lymphocytes and neutrophils infiltrate around acne inflammatory lesions (27), lymphocytes may play a more central role in early acne lesions than neutrophils, which are strongly attracted after the follicles have been disrupted (28). Additionally, other inflammatory cells, especially CD4^+^ T cells and macrophages, are also observed in the perifollicular region and dermis in acne-uninvolved skin (10). This line of evidence suggests the involvement of innate and adaptive immune processes in the pathogenesis of acne vulgaris. Further studies indicate that acnes at early stage, 6-72 hours after the development of lesions, only show small papules with a minimal erythema, with neither rupture of the follicular walls nor neutrophilic infiltration. After 72 hours of the development of acne, neutrophils can be observed in 33% of lesions (28). This evidence indicates that acne vulgaris is featured by microcomedones and small papules in early stage, followed by neutrophilic infiltration. There is no agreed definition of the early stages of acne vulgaris. We defined microcomedones and small papules with no disruption of the follicle wall as the early stage of acne in our review (**Figure 1**).

## 3 Adaptive immune cells

### 3.1 T helper 1 cells

Epidermal T cells, mainly CD8^+^ T cells, are distributed in the stratum basale and stratum spinosum, while dermal T cells are often situated beneath the dermal-epidermal junction or adjacent to cutaneous appendages (29). The number of CD4^+^ T cells in the epidermis is comparable to that in the dermis, and they are only found around hair follicles. Under physiological conditions, 98% of cutaneous lymphocyte-associated antigen (CLA)^+^ effector memory T cells reside in the skin and can initiate and perpetuate immune reactions without recruiting T cells from the blood (30). CD4^+^ T helper (Th) cells regulate adaptive immune responses by secreting cytokines and chemokines to activate and recruit effector cells (31).

Previous studies showed that a subpopulation of *C. acnes*-specific Th1 cells is present in early acne lesions, while *C. acnes* can stimulate T cell proliferation (32, 33). Acne lesions exhibit high expression levels of Th1 effector cytokine interferon-γ (IFN-γ), Th1 polarizing key transcription factor T-bet, and the pivotal Th1 activating cytokine interleukin 12 (IL-12), suggesting the role of Th1 cells in acne. (33, 34). *C. acnes* induces production of IL-12 by monocytes via Toll-like receptor-2 (TLR-2) signaling. The innate immune system recognizes *C. acnes* via TLR-2, increasing the levels of IL-8 and IL-12 (35). In turn, IL-12 activates the transcription factor signal transducer and activator of transcription 4 (STAT4), inducing the production of IFN-γ by Th1 cells (36), while IFN-γ promotes the differentiation of Th1 cells and induces chemokine secretion to recruit immune cells. IFN-γ–stimulated sebocytes seem to foster the migration of CD45RO^+^ T cells with no influence on cytokine secretion (37).

### 3.2 T helper 17 cells

In comparison to the skin of healthy individuals, acne-involved skin displays a high number of IL-17^+^ cells near the PSU (34, 38, 39). The dermal IL-17^+^ cells are lymphocytes, which affect epidermal keratinocytes in a paracrine manner (40). There is a significant elevation in Th17 lineage signature cytokines, including IL-1β, IL-6 and transforming growth factor-β (TGF-β), in acne lesional vs. nonlesional skin (34). *C. acnes* increases expression levels of key Th17-related genes in human peripheral blood mononuclear cells (PBMCs) (38). Correspondingly, an integrated bioinformatics study demonstrates increased infiltration of Th17 cells and Th17-related cytokines in acne lesions (41). Moreover, the number of Th17 cells is increased in the closed comedone stage of acne, indicating that Th17 cells are involved in the pathogenesis of acne, at least in early stage (42). 

Sebocytes can drive a Th17 immune response via the production of IL-6, TGF-β and IL-1β. Sebocytes can recruit various subsets of T cells, including CD4^+^CD45RO^+^ effector and CD4^+^CD45RA^+^ naive T cells in a CXCL8-dependent manner. Although sebocytes do not alter the effector T-cell phenotype, they affect the migration of naive T cells and alter their developmental trajectory towards Th17 cells via the secretion of IL-6, TGF-β and IL-1β (37). In addition to its effects on Th17 cells, *C. acnes* can also promote mixed Th17/Th1 cell and Th1-like cell responses *in vitro* by inducing concomitant secretion of IL-17A and IFN-γ (39). These mixed Th17/Th1 cytokines are most likely derived from Th17 subsets displaying a degree of plasticity and acquiring functional characteristics of Th1 cells (43). Acne-associated *C. acnes* strains provide a microbial microenvironment, regulating the programs responsible for the differentiation of Th17 cells into Th17/Th1 cells (44).

Th17 cells are characterized by the production of IL-17A and IL-17F and potent inducers of tissue inflammation. IL-17 and IL-22, effector cytokines of Th17 cells, enhance the expression of antimicrobial peptides (AMPs), including cathelicidins and β-defensins (45). Human β-defensin-2 (hBD)-2 is elevated in acne. AMPs suppress excess cytokine release after minor epidermal injury to maintain inflammatory homeostasis. Other studies also showed that AMPs promote additional inflammatory responses in addition to their antibacterial activity (46, 47). Although Th17 cells can strengthen the body’s defense against extracellular pathogens, the excessive Th17 responses can drive chronic inflammation, likely contributing to the development of acne (48). The role of Th17 response in acne cannot be dissociated from the local microenvironment, i.e., dysseborrhea and loss of *C. acnes* phylotype diversity.

While Th17-cell-derived IL-26 exerts direct antimicrobial activity against extracellular bacteria, it lacks antimicrobial potency against *C. acnes* (44, 49). *C. acnes* phylotypes directly influence the Th17 cytokine profile and differentially modulate the CD4^+^ T cell responses involving the generation of Th17 cells. *C. acnes* phylotypes IA2, IB, and IC are increased in acne patients. The acne-related *C. acnes* subtypes increase secretion of IFN-γ and IL-17, while decreasing levels of IL-10 in PBMCs. In contrast, healthy skin-related *C. acnes* subtypes increase IL-10 levels (50). IL-10 can repress proinflammatory responses by downregulating IFN-γ and IL-17 (51). IL-10-producing Th17 cells are protective and exhibit microbicidal activity against *C. acnes*, whereas IFN-γ-producing Th17 cells are pathogenic without microbicidal activity (44). Acne-associated *C. acnes* strains promote the differentiation of a non-antimicrobial Th17 subpopulation (n-_AM_Th17). Healthy skin-related *C. acnes* strains can specifically stimulate antimicrobial subpopulation of Th17 cells (_AM_Th17) to secrete antimicrobial proteins and generate T-cell extracellular traps (TETs) capable of capturing and killing *C. acnes*. *C. acnes* is entangled in TETs in proximity to Th17 cells in acne lesional skin (52). Although TETs are involved in antimicrobial responses, whether TETs exacerbate inflammation is unclear.

### 3.3 Regulatory T cells

In inflammatory disorders, Th17 cells have intimate links with Foxp3-expressing regulatory T (Treg) cells in immune balance. Tissue-resident Treg cells are predominantly distributed near the hair bulge area in the steady state (53). Tregs are efficient suppressors of both innate and adaptive immune responses, which are well known to be involved in preservation of cutaneous homeostasis and in the regulation of skin immune response (54). Significantly high numbers of Foxp3^+^ cells are observed in the papillary dermis in early acne lesions (34, 40). Treg cells in acne patients may have functional deficiency to suppress the abnormality persistent immune response in acne lesions. Treg cells lose their suppressive function and become IL-17-expressing cells under inflammatory conditions. The dysfunction of Treg cells might be a underlying mechanism accounting for chronic skin inflammation (55–57). Moreover, the number of Tregs is lower in acne lesions than in nonlesional skin of acne patients (41). However, whether an increase in the number of Treg cells alone can benefit acne remains to be determined.

Immunopathogenesis of acne vulgaris may be related to deviations of the Th17/Treg balance (41). Increases in the Th17/Treg ratio may contribute to the initiation of inflammatory processes and can negatively affect Treg-controlled homeostasis and integrity of hair follicles (58). Retinoids exert beneficial effects on acne, via inhibition of IL-17 and increase in Foxp3 expression, whereby regulating the balance between Treg and Th17 cell differentiation (59, 60). The effective drugs treatment should not only attenuate Th17/IL-17 signaling, but also improve Treg function in order to stabilize the hair follicles. Comparison of the ratio of Th17/Treg cells between acne lesional skin and healthy skin and clarification of Treg-related disturbances of homeostasis of hair follicle would be helpful to elucidate the pathogenesis of acne vulgaris.

## 4 Innate immune cells

### 4.1 Dendritic cells

Dendritic cells (DCs) are a family of antigen-sensing and antigen-presenting cells that link the innate and adaptive immune systems (61). Skin DCs can be classified into four types: epidermal Langerhans cells (LCs), conventional DCs (cDCs), plasmacytoid DCs (pDCs) and monocyte-derived DCs (62). DC subsets are developmentally imprinted and modulated by local microenvironmental and inflammatory state (63). LCs are the main DC subsets in the epidermis, taking up and processing antigens for presentation to skin resident memory T cells or effector T cells (64, 65).

Skin immunohistochemistry revealed that CD1^+^cells (considered to be LCs) and CD83^+^ dendritic cells were significantly higher in early acne stage than in nonlesional skin (10, 27, 28, 34). An analysis of skin biopsy samples also noted a clear increase in the number of LCs and DCs in the closed comedone stage. Interestingly, cDC2s are associated with perilesional CD4^+^T cells (42). Bacterial peptidoglycan (PGN)-activated DCs selectively produce IL-1 and IL-23, which efficiently activate protective Th17 cells (66, 67). It has been postulated that changes in the follicular microenvironment may increase the production of immunogenic *C. acnes* proteins. LCs process antigens and migrate to the local lymph node, where antigens are presented to CD4^+^T cells (68).

### 4.2 Macrophages

Macrophages are usually regarded as terminally differentiated monocytic phagocytes. Monocytes are recruited to the tissue where they differentiate into macrophages. Macrophages are activated by different stimuli and exert heterogeneous effects in healthy and inflamed skin, and based on these effects, they can be classified into classically (M1) and alternatively (M2) activated subsets (69, 70).

Number of CD68^+^ macrophages is significantly higher both in early acne lesions and uninvolved follicles in acne patients compared with healthy subject (10, 34, 42). *C. acnes* triggers inflammatory cytokine expression through the activation of TLR2 on macrophages, followed by the activation of the NOD-like receptor thermal protein domain associated protein 3 (NLRP3) inflammasome, Nuclear factor kappa-B (NF- κB) as well as mitogen-activated protein kinase (MAPK) signaling cascade (71–74). TLR2^+^ macrophages are present in acne lesions and increased during the evolution of the disease (35). *C. acnes* can also stimulate type I interferon (IFN-I) synthesis via the wiring of a TLR2- TIR-domain-containing adapter-inducing interferon-β (TRIF) pathway in human macrophages (75). In addition, IFN-I stimulates and amplifies the secretion of chemokines and other immune mediators, contributing to inflammatory responses (76).

Under normal conditions, M1 macrophages, also termed as skin-resident macrophages, surround the sebaceous glands (77, 78). Both M1 and M2 subsets can be found in acne lesions (79), and M1-like macrophages mount an antimicrobial response against *C. acnes (* 80). Sebum can affect the polarization of macrophages favoring the generation of M2 macrophages (81). Lipids that accumulate in the PSU are oxidized by *C. acnes* lipase, and macrophages can phagocytose oxidized lipids, consequently becoming foam cells (82). These foam cells express TREM2 and infiltrate in acne lesions. The sebum of acne patients has a higher content of squalene (83), which can increase TREM2 expression on macrophages. TREM2 expression enhances the phagocytic capacity of the macrophages to uptake lipids and bacteria, but these macrophages are unable to kill the bacteria. Squalene-induced TREM2 macrophages contribute to inflammation by up-regulating expression of proinflammatory chemokines, cytokines, MMPs, and S100 proteins to recruit and activate immune cells (79). Accumulation of intracellular lipids and lipid metabolic products trigger the production of proinflammatory cytokines in macrophages, contributing to the immunopathology of early acne vulgaris. Notably, TREM2 macrophages are not typically present in other inflammatory skin diseases, such as psoriasis (84) and atopic dermatitis (85). However, the pathogenic role of macrophages in acne has not been fully elucidated yet and more studies are needed to characterize the functional of macrophage in acne.

### 4.3 Mast cells

Mast cells (MCs) are most abundant in the upper dermis and are located near blood vessels and nerve endings under physiological conditions. The MC number is not affected by age or sex (86). MCs are key effector cells that respond to allergic inflammation and innate immune responses against bacteria. A number of factors can activate MCs to release granule-stored mediators and synthesize other types of mediators, leading to the development of inflammatory dermatoses (87).

The high-affinity IgE receptor (FcϵRI) and CD69 are strongly expressed in acne lesions (42). MC number and CD69 expression peaked in the closed comedone stage, indicating that activated MCs are involved in early acne lesions. The increase in the number of MCs depends on keratinocyte-produced stem cell factor (SCF). Lipoteichoic acid (LTA), a gram-positive cell wall component, stimulates an increase in the production of SCF in keratinocytes, indirectly influencing the recruitment and maturation of MCs (88). A colocalization experiment showed that most IL-17A^+^ cells are positive for tryptase (a MC marker) and negative for CD3 and CD4, markers of T cells. Thus, MCs are possibly the cellular source of IL-17A rather than CD4^+^ T cells in closed comedone (42). Activated Th cells drive IL-17A production in MCs via cell-cell contact. Neither classical MC stimuli nor Th cell cytokines induce IL-17 production in MCs, which means the mechanism underlying IL-17 production by MCs is tightly regulated (42, 89). IL-17A, a proinflammatory cytokine, increases CXC ligand (CXCL)8 production in epithelial cells and activates fibroblasts to recruit neutrophils (90), while neutrophils generate reactive oxygen species (ROS) that irritate and destroy follicular integrity, causing inflammatory progression of acne lesions, which are then classified as pustules (91, 92). Moreover, IL-17A synergizes with other inflammatory cytokines, leading to increased production of IL-6 and IL-8 (93). IL-17 is not a typical mast cell cytokine, but it is increasingly appreciated that innate immune cells can produce IL-17 during an inflammatory response (94). However, the underlying mechanisms by which mast cells secrete IL-17 are not clear. To understand the complex pathophysiology of acne vulgaris, it is imperative to define the mechanisms mediating IL-17 release.

### 4.4 Innate lymphoid cells

Innate lymphoid cells (ILCs) exhibit a lymphoid morphology; they do not express rearranged antigen-specific receptors but do have important functions in innate immunity and tissue remodeling. ILCs are subdivided into 3 subsets, ILC1s, ILC2s and ILC3s. ILC2s are the predominant tissue-resident skin ILC subset under steady state and during inflammation (95, 96). Lack of ILCs causes sebaceous hyperplasia and alters the equilibrium of skin commensal bacteria by modulating the production of palmitoleic acid, a component of sebum with antimicrobial properties, and inhibiting the growth of several species of gram-positive cocci (97). Sebaceous hyperplasia and dyshomeostasis of skin commensal bacteria induce inflammation in the pathogenesis of acne vulgaris, which means that ILCs may be involved in the early stage of inflammation in acne. A large number of ILC3s are present in the non-lesional skin in hidradenitis suppurativa (HS) (98). Both IL-1β and IL-23 can activate ILC3s to produce IL-22 and IL-17 (99, 100). With expression of multiple Th17- and Th1-derived cytokines, ILCs are subsequently replaced by adaptive Th mediated response. It remains to be seen whether ILCs operate in the same way in humans as they do in experimental animal models. Future study is needed to investigate ILC subsets in skin of patients with acne and characterize the functional capacity of ILC to contribute to immune responses.

## 5 Skin cells involved in acne inflammation

### 5.1 Keratinocytes

As the major cell type in the epidermis, keratinocytes not only form a physical barrier but also secrete cytokines to modulate the immune response and inflammation (101). Keratinocytes express different types of pattern recognition receptors (PRRs), recognizing various pathogens and secreting cytokines, chemokines, and AMPs (102). Keratinocytes constitutively synthesize IL-1α and IL-1β (103). Excessive skin colonization of *C. acnes* can activate TLR-2 and TLR-4 on keratinocytes, resulting in the production of a panel of inflammatory mediators, including IL-8, IL-6, IL-1α, TNF-α, granulocyte–macrophage colony-stimulating factor (GM-CSF), matrix metalloproteinase (MMP)-9 and hBD-2 (74, 104–107). These mediators activate tissue-resident immune cells to induce and perpetuate an inflammatory response. *C. acnes* is also recognized by CD36, a scavenger receptor expressed on keratinocytes, inducing a rapid production of ROS by keratinocytes, consequently leading to inhibition of bacterial growth and production of inflammation (108). Moreover, keratinocytes in hair follicles express squalene epoxidase, which converts squalene to squalene epoxide (79). Lipid peroxides, in particular squalene peroxides, have been shown to activate lipoxygenases and increase the production of IL-6 in keratinocytes in a dose-dependent manner (109). In addition, hypoxia due to increasing intraductal pressure may induce hypoxia inducible factor (HIF)-1 production, stimulating keratinocytes to produce proinflammatory cytokines (24, 110). Thus, keratinocytes can contribute at least in part to the inflammation in acne vulgaris.

### 5.2 Sebocytes

Sebocytes form the sebaceous gland acini belonging to the upper PSU (111, 112). Matured sebocytes secrete their contents in a holocrine manner, leading to DNase2-mediated programmed cell death (113), which affects skin barrier function (114). Human sebum is a lipid mixture, and wax esters and squalene are characteristic of sebocytes (115, 116). Sebocytes may act as immune-active cells, recognizing microorganisms and then producing AMPs and cytokines. Sebocytes are not only a target of inflammation, but also modulate of immunity (117, 118). Increased activity of androgen hormones and insulin-like growth factor 1 (IGF-1) stimulates the proliferation and differentiation of sebocytes, resulting in hyperseborrhea (119). Clinical research has demonstrated a positive correlation between serum IGF-1 levels and disease severity, especially in female acne patients (120). IGF-1 induces the expression of proinflammatory cytokines, such as IL-1β, IL-6, IL-8, and TNF-α, in sebocytes via the NF-κB signaling pathway (121). Sebocytes express PRRs, such as TLR2, TLR4, TLR6 and CD14, to recognize *C. acnes* and produce IL-1β, IL-6 and TGF-β *in vitro*, which drives a Th17 immune response (37, 122–125). GATA6 expressed in differentiating sebocytes can induce the expression of IL-10 and negatively regulates acne-driven IL-8 and IL-17 cytokines. Expression levels of GATA6 are reduced in early acne lesions, resulting in increased acne-driven cytokines (126).

Bacterial lipases hydrolyze some of the triglycerides in the sebum to free fatty acids (FFAs), which have a proinflammatory effect and antibacterial activity (127, 128). Proteases produced by *C. acnes* activate protease-activated receptor-2 (PAR-2) on sebocytes can also induce the production of inflammatory cytokines and antimicrobial peptides (129). FFAs and *C. acnes* upregulate the expression of hBD-2 in human sebocytes to enhance innate immune defense (47, 130). The development of more anaerobic conditions in hair follicles can lead to outgrowth of *C. acnes* and buildup of short-chain fatty acids (SCFAs) (131, 132). SCFAs have been shown to amplify TLR-driven cytokine responses from sebocytes through inhibition of histone deacetylase activity and the activation of fatty acid receptors (132).

Moreover, sebocytes secrete biologically active lipids to regulate inflammation. Sebum from acne patients contains lower levels of linoleic acid and higher levels of squalene, lipoperoxides, and monounsaturated fatty acids (MUFAs), particularly palmitoleic acid (C16:1) and oleic acid (C18:1) (83, 133–135). Stearoyl-CoA desaturase (SCD) and fatty acid desaturase (FADS)-2, two enzymes responsible for the biosynthesis of MUFAs in sebocytes, are upregulated by the TLR-2 ligand macrophage-activating lipopeptide-2 (MALP2) (122, 136). Excessive generation of squalene and MUFAs increases the rate of lipid peroxidation, and their oxidation products create a proinflammatory environment and induce comedogenesis (135, 137). Palmitic acid activates the NLRP3 inflammasome to induce release of IL-1β (138) and inflammatory response in sebocytes via TLR2 and TLR4 signaling (128). Epidermal growth factor together with palmitic acid may augment the inflammatory properties of sebocytes (139). In contrast, linoleic acid has an anti-inflammatory effect via inhibition of IL-1β production in *C. acnes*-activated macrophages (81). It is qualitative changes, not quantitative changes, in sebum composition that play a central role in the development of acne (26). Finally, sebocytes can release leptin after being triggered by TLR-2 and TLR-4 or mTORC1 pathway (118, 140). Sebocyte-derived leptin induces the expression of proinflammatory lipids, such as cyclooxygenase 2 (COX-2) and 5-lipoxygenase (5-LOX), and augments the expression of IL-6 and IL-8 (141, 142). Leptin also plays a pivotal role in Th17 cell differentiation (143). Sebocytes expressing leptin receptor (LEPR) may perpetuate inflammation in an autocrine manner (144). Collectively, sebocytes can provoke inflammation in acne via multiple mechanisms.

### 5.3 Fibroblasts

Dermal fibroblasts are essential cells that support the structural integrity of tissues. Dermal white adipose tissue (dWAT) is a unique tissue layer made up of adipocytes mainly concentrated around the PSUs (145). Intradermal infection with *Staphylococcus aureus* induces proliferation and differentiation of fibroblasts into the preadipocyte lineage, leading to rapid expansion of the dWAT layer and triggering the production of antimicrobial peptides, a process dubbed reactive adipogenesis (146). Recent studies have shown that reactive adipogenesis occurs in the perifollicular stroma of acne. *C. acnes* triggers dermal fibroblast differentiation and enhances cathelicidin expression, which is partially dependent on TLR2 activity (147). Hence, dermal perifollicular fibroblasts are involved in the pathogenesis of acne and represent a potential target for acne therapy.

## 6 Conclusions

Acne lesions begin with the formation of microcomedones. Follicular epidermal hyperproliferation, increased sebum production and the growth of *C. acnes* in PSUs contribute to microcomedone formation.

**Figure 2:**
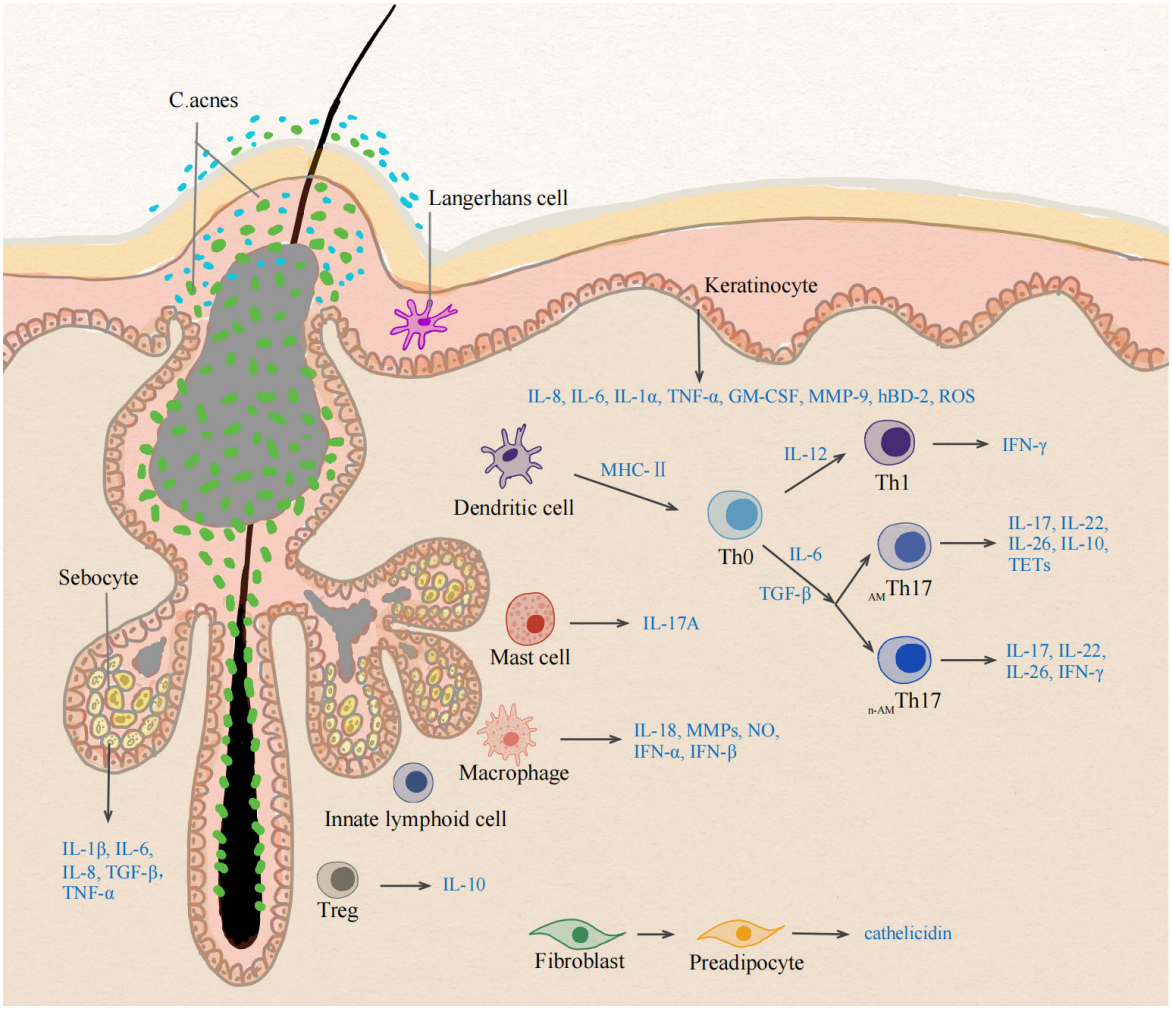
Different cell types at the early stage of inflammation in acne vulgaris. The early stage of acne vulgaris manifests microcomedones and small papules, which has no disruption of the follicle wall. The change of follicle microenvironment in acne initiate the immune activation of skin cells. Activated sebocytes, keratinocytes and skin-resident APCs upregulate the production of pro-inflammatory mediators, such as IL-1β, IL-6, IL-12 and TGF-β. IL-6 and TGF-β induce the differentiation into Th17 cells, whereas IL-12 drives a Th1 differentiation program. Healthy-related *C. acnes* induce IL-10-producing _AM_Th17 cells, whereas acne-associated strains promote the development of n-_AM_Th17 cells. _AM_Th17 cells release IL-17, IL-22, IL-26, IL-10 and TETs, n-_AM_Th17 cells induce IFN-γ. Treg lose their suppressive function for deviations of the Th17/Treg balance. MCs are the cellular source of IL-17A in early acne. Lack of ILCs leads to sebaceous hyperplasia and alters the equilibrium of skin commensal bacteria. Accumulation of intracellular lipids and lipid metabolic products induce the production of proinflammatory cytokines in macrophages. *C. acnes* triggers dermal fibroblast differentiation and enhances cathelicidin expression. APC, Antigen presenting cell; TGF-β, Transforming growth factor- Beta; IFN-γ, Interferon gamma; MC, mast cell; TETs, T-cell extracellular traps; ILCs, innate lymphoid cells. _AM_Th17 cells, antimicrobial Th17 cells; n-_AM_Th17 cells, non-antimicrobial Th17 cells.

The alteration of the follicle microenvironment stimulates skin-resident antigen presenting cells (APCs), sebocytes, and keratinocytes to produce proinflammatory cytokines, such as IL-1β, IL-6, and TGF-β. Macrophages phagocytose oxidized lipids and produce proinflammatory cytokines. MCs appeared as pioneer cells to produce IL-17, followed by the appearance of ILCs and Th cells. With the expression of multiple Th17- and Th1-derived cytokines, adaptive Th-mediated response plays a pivotal role in the early stage of acne. Deviations of the Th17/Treg balance may contribute to the initiation of inflammatory processes and negatively affect PSU homeostasis destabilizing the hair follicle infundibulum (**Figure 2**). The follicle walls eventually rupture, and neutrophils take over, increasing the latter stage of IL-17 production and triggering a rapid inflammatory response. The crosstalk of different skin cells in the early stage of acne remains to be revealed. Understanding these skin immune cells in the pathogenesis of early acne can facilitate the identification of biomarkers as well as the development of targeted therapies for acne vulgaris. Because of immune overactivation in acne, anti-inflammatory treatments should be employed in the management of acne.

## Author contributions

LH: Writing – original draft, Writing – review & editing. SY: Writing – original draft, Writing – review & editing. XY: Writing – review & editing. FF: Writing – review & editing. LZ: Writing – review & editing. LW: Writing – review & editing. XZ: Writing – review & editing. CY: Writing – review & editing. QQ: Writing – review & editing. TZ: Writing – original draft, Writing – review & editing.

## References

[1] Vos T Flaxman AD Naghavi M Lozano R Michaud C Ezzati M. Years lived with disability (YLDs) for 1160 sequelae of 289 diseases and injuries 1990-2010: a systematic analysis for the Global Burden of Disease Study 2010. Lancet (London England) (2012) 380(9859):2163–96 doi: 10.1016/S0140-6736(12)61729-2. PMC6350784 23245607

[2] Tan JKL Bhate K. A global perspective on the epidemiology of acne. Br J Dermatol (2015) 172 Suppl 1:3–12. doi: 10.1111/bjd.13462 25597339

[3] Layton AM. Optimal management of acne to prevent scarring and psychological sequelae. Am J Clin Dermatol (2001) 2(3):135–41. doi: 10.2165/00128071-200102030-00002 11705090

[4] Schneider MR Paus R. Deciphering the functions of the hair follicle infundibulum in skin physiology and disease. Cell Tissue Res (2014) 358(3):697–704. doi: 10.1007/s00441-014-1999-1 25248789

[5] Do TT Zarkhin S Orringer JS Nemeth S Hamilton T Sachs D. Computer-assisted alignment and tracking of acne lesions indicate that most inflammatory lesions arise from comedones and de novo. J Am Acad Dermatol (2008) 58(4):603–8. doi: 10.1016/j.jaad.2007.12.024 18249468

[6] Moradi Tuchayi S Makrantonaki E Ganceviciene R Dessinioti C Feldman SR Zouboulis CC. Acne vulgaris. Nat Rev Dis Primers. (2015) 1:15029. doi: 10.1038/nrdp.2015.29 27189872

[7] Gollnick H Cunliffe W Berson D Dreno B Finlay A Leyden JJ. Management of acne: a report from a Global Alliance to Improve Outcomes in Acne. J Am Acad Dermatol (2003) 49(1 Suppl):S1–37. doi: 10.1067/mjd.2003.618 12833004

[8] Cong TX Hao D Wen X Li XH He G Jiang X. From pathogenesis of acne vulgaris to anti-acne agents. Arch Dermatol Res (2019) 311(5):337–49. doi: 10.1007/s00403-019-01908-x 30859308

[9] Saurat JH. Strategic targets in acne: the comedone switch in question. Dermatology (2015) 231(2):105–11. doi: 10.1159/000382031 26113292

[10] Jeremy AHT Holland DB Roberts SG Thomson KF Cunliffe WJ. Inflammatory events are involved in acne lesion initiation. J Invest Dermatol (2003) 121(1):20–7. doi: 10.1046/j.1523-1747.2003.12321.x 12839559

[11] Dréno B Pécastaings S Corvec S Veraldi S Khammari A Roques C. *Cutibacterium acnes (Propionibacterium acnes)* and *acne vulgaris*: a brief look at the latest updates. J Eur Acad Dermatol Venereol. (2018) 32 Suppl 2:5–14. doi: 10.1111/jdv.15043 29894579

[12] Szabó K Erdei L Bolla BS Tax G Bíró T Kemény L. Factors shaping the composition of the cutaneous microbiota. Br J Dermatol (2017) 176(2):344–51. doi: 10.1111/bjd.14967 27518483

[13] Dréno B Dagnelie MA Khammari A Corvec S. The skin microbiome: A new actor in inflammatory acne. Am J Clin Dermatol (2020) 21(Suppl 1):18–24. doi: 10.1007/s40257-020-00531-1 32910436 PMC7584556

[14] McDowell A Valanne S Ramage G Tunney MM Glenn JV McLorinan GC. *Propionibacterium acnes* types I and II represent phylogenetically distinct groups. J Clin Microbiol (2005) 43(1):326–34. doi: 10.1128/JCM.43.1.326-334.2005 PMC540145 15634990

[15] McDowell A Perry AL Lambert PA Patrick S. A new phylogenetic group of Propionibacterium acnes. J Med Microbiol (2008) 57(Pt 2):218–24. doi: 10.1099/jmm.0.47489-0 18201989

[16] McDowell A Barnard E Nagy I Gao A Tomida S Li H. An expanded multilocus sequence typing scheme for *propionibacterium acnes*: investigation of ‘pathogenic’, ‘commensal’ and antibiotic resistant strains. PloS One (2012) 7(7):e41480. doi: 10.1371/journal.pone.0041480 22859988 PMC3408437

[17] McDowell A Gao A Barnard E Fink C Murray PI Dowson CG. A novel multilocus sequence typing scheme for the opportunistic pathogen *Propionibacterium acnes* and characterization of type I cell surface-associated antigens. Microbiol (Reading). (2011) 157(Pt 7):1990–2003. doi: 10.1099/mic.0.049676-0 21511767

[18] Josse G Mias C Le Digabel J Filiol J Ipinazar C Villaret A. High bacterial colonization and lipase activity in microcomedones. Exp Dermatol (2020) 29(2):168–76. doi: 10.1111/exd.14069 PMC7586799 31863492

[19] Dreno B Gollnick HP Kang S Thiboutot D Bettoli V Torres V. Understanding innate immunity and inflammation in acne: implications for management. J Eur Acad Dermatol Venereology: JEADV (2015) 29 Suppl 4:3–11. doi: 10.1111/jdv.13190 26059728

[20] Blakney AK McKay PF Ibarzo Yus B Hunter JE Dex EA Shattock RJ. The skin you are in: design-of-experiments optimization of lipid nanoparticle self-amplifying RNA formulations in human skin explants. ACS Nano. (2019) 13(5):5920–30. doi: 10.1021/acsnano.9b01774 PMC7007275 31046232

[21] Byrd AL Belkaid Y Segre JA. The human skin microbiome. Nat Rev Microbiol (2018) 16(3):143–55. doi: 10.1038/nrmicro.2017.157 29332945

[22] Kabashima K Honda T Ginhoux F Egawa G. The immunological anatomy of the skin. Nat Rev Immunol (2019) 19(1):19–30. doi: 10.1038/s41577-018-0084-5 30429578

[23] Zhang C Merana GR Harris-Tryon T Scharschmidt TC. Skin immunity: dissecting the complex biology of our body’s outer barrier. Mucosal Immunol (2022) 15(4):551–61. doi: 10.1038/s41385-022-00505-y 35361906

[24] Danby FW. Ductal hypoxia in acne: is it the missing link between comedogenesis and inflammation? J Am Acad Dermatol (2014) 70(5):948–9. doi: 10.1016/j.jaad.2013.11.029 24742839

[25] Choi K Jin M Zouboulis CC Lee Y. Increased lipid accumulation under hypoxia in SZ95 human sebocytes. Dermatology (2021) 237(1):131–41. doi: 10.1159/000505537 32088721

[26] Zouboulis CC Jourdan E Picardo M. Acne is an inflammatory disease and alterations of sebum composition initiate acne lesions. J Eur Acad Dermatol Venereol. (2014) 28(5):527–32. doi: 10.1111/jdv.12298 24134468

[27] Layton AM Morris C Cunliffe WJ Ingham E. Immunohistochemical investigation of evolving inflammation in lesions of acne vulgaris. Exp Dermatol (1998) 7(4):191–7. doi: 10.1111/j.1600-0625.1998.tb00323.x 9758417

[28] Norris JF Cunliffe WJ. A histological and immunocytochemical study of early acne lesions. Br J Dermatol (1988) 118(5):651–9. doi: 10.1111/j.1365-2133.1988.tb02566.x 2969256

[29] Bos JD Zonneveld I Das PK Krieg SR van der Loos CM Kapsenberg ML. The skin immune system (SIS): distribution and immunophenotype of lymphocyte subpopulations in normal human skin. J Invest Dermatol (1987) 88(5):569–73. doi: 10.1111/1523-1747.ep12470172 3494791

[30] Clark RA Chong B Mirchandani N Brinster NK Yamanaka KI Dowgiert RK. The vast majority of CLA+ T cells are resident in normal skin. J Immunol (2006) 176(7):4431–9. doi: 10.4049/jimmunol.176.7.4431 16547281

[31] Zhu J Yamane H Paul WE. Differentiation of effector CD4 T cell populations (*). Annu Rev Immunol (2010) 28:445–89. doi: 10.1146/annurev-immunol-030409-101212 PMC3502616 20192806

[32] Jappe U Ingham E Henwood J Holland KT. *Propionibacterium acnes* and inflammation in acne; P. acnes has T-cell mitogenic activity. Br J Dermatol (2002) 146(2):202–9. doi: 10.1046/j.1365-2133.2002.04602.x 11903228

[33] Mouser PE Baker BS Seaton ED Chu AC. *Propionibacterium acnes*-reactive T helper-1 cells in the skin of patients with acne vulgaris. J Invest Dermatol (2003) 121(5):1226–8. doi: 10.1046/j.1523-1747.2003.12550_6.x 14708633

[34] Kelhälä HL Palatsi R Fyhrquist N Lehtimäki S Väyrynen JP Kallioinen M. IL-17/Th17 pathway is activated in acne lesions. PloS One (2014) 9(8):e105238. doi: 10.1371/journal.pone.0105238 25153527 PMC4143215

[35] Kim J. Review of the innate immune response in acne vulgaris: activation of Toll-like receptor 2 in acne triggers inflammatory cytokine responses. Dermatology (2005) 211(3):193–8. doi: 10.1159/000087011 16205063

[36] Guo L Wei G Zhu J Liao W Leonard WJ Zhao K. IL-1 family members and STAT activators induce cytokine production by Th2, Th17, and Th1 cells. Proc Natl Acad Sci U S A. (2009) 106(32):13463–8. doi: 10.1073/pnas.0906988106 PMC2726336 19666510

[37] Mattii M Lovászi M Garzorz N Atenhan A Quaranta M Lauffer F. Sebocytes contribute to skin inflammation by promoting the differentiation of T helper 17 cells. Br J Dermatol (2018) 178(3):722–30. doi: 10.1111/bjd.15879 28799643

[38] Agak GW Qin M Nobe J Kim MH Krutzik SR Tristan GR. *Propionibacterium acnes* induces an IL-17 response in acne vulgaris that is regulated by vitamin A and vitamin D. J Invest Dermatol (2014) 134(2):366–73. doi: 10.1038/jid.2013.334 PMC4084940 23924903

[39] Kistowska M Meier B Proust T Feldmeyer L Cozzio A Kuendig T. *Propionibacterium acnes* promotes Th17 and Th17/Th1 responses in acne patients. J Invest Dermatol (2015) 135(1):110–8. doi: 10.1038/jid.2014.290 25010142

[40] Farag AGA Maraee AH Rifaat Al-Sharaky D Elshaib ME Kohla MSM Shehata WA. Tissue expression of IL-17A and FOXP3 in acne vulgaris patients. J Cosmet Dermatol (2021) 20(1):330–7. doi: 10.1111/jocd.13485 32413182

[41] Yang L Shou YH Yang YS Xu JH. Elucidating the immune infiltration in acne and its comparison with rosacea by integrated bioinformatics analysis. PloS One (2021) 16(3):e0248650. doi: 10.1371/journal.pone.0248650 33760854 PMC7990205

[42] Eliasse Y Leveque E Garidou L Battut L McKenzie B Nocera T. IL-17+ Mast cell/T helper cell axis in the early stages of acne. Front Immunol (2021) 12:740540. doi: 10.3389/fimmu.2021.740540 34650562 PMC8506309

[43] Muranski P Restifo NP. Essentials of Th17 cell commitment and plasticity. Blood (2013) 121(13):2402–14. doi: 10.1182/blood-2012-09-378653 PMC3612853 23325835

[44] Agak GW Kao S Ouyang K Qin M Moon D Butt A. Phenotype and antimicrobial activity of th17 cells induced by *propionibacterium acnes* strains associated with healthy and acne skin. J Invest Dermatol (2018) 138(2):316–24. doi: 10.1016/j.jid.2017.07.842 PMC5794628 28864077

[45] Liang SC Tan XY Luxenberg DP Karim R Dunussi-Joannopoulos K Collins M. Interleukin (IL)-22 and IL-17 are coexpressed by Th17 cells and cooperatively enhance expression of antimicrobial peptides. J Exp Med (2006) 203(10):2271–9. doi: 10.1084/jem.20061308 PMC2118116 16982811

[46] Harder J Tsuruta D Murakami M Kurokawa I. What is the role of antimicrobial peptides (AMP) in acne vulgaris? Exp Dermatol (2013) 22(6). doi: 10.1111/exd.12159 23711061

[47] Chronnell CM Ghali LR Ali RS Quinn AG Holland DB Bull JJ. Human beta defensin-1 and -2 expression in human pilosebaceous units: upregulation in acne vulgaris lesions. J Invest Dermatol (2001) 117(5):1120–5. doi: 10.1046/j.0022-202x.2001.01569.x 11710922

[48] Mias C Mengeaud V Bessou-Touya S Duplan H. Recent advances in understanding inflammatory acne: Deciphering the relationship between Cutibacterium acnes and Th17 inflammatory pathway. J Eur Acad Dermatol Venereol. (2023) 37 Suppl 2:3–11. doi: 10.1111/jdv.18794 36729400

[49] Meller S Di Domizio J Voo KS Friedrich HC Chamilos G Ganguly D. T(H)17 cells promote microbial killing and innate immune sensing of DNA via interleukin 26. Nat Immunol (2015) 16(9):970–9. doi: 10.1038/ni.3211 PMC4776746 26168081

[50] Yu Y Champer J Agak GW Kao S Modlin RL Kim J. Different *propionibacterium acnes* phylotypes induce distinct immune responses and express unique surface and secreted proteomes. J Invest Dermatol (2016) 136(11):2221–8. doi: 10.1016/j.jid.2016.06.615 PMC8295477 27377696

[51] Ouyang W Rutz S Crellin NK Valdez PA Hymowitz SG. Regulation and functions of the IL-10 family of cytokines in inflammation and disease. Annu Rev Immunol (2011) 29:71–109. doi: 10.1146/annurev-immunol-031210-101312 21166540

[52] Agak GW Mouton A Teles RM Weston T Morselli M Andrade PR. Extracellular traps released by antimicrobial TH17 cells contribute to host defense. J Clin Invest. (2021) 131(2):141594. doi: 10.1172/JCI141594 33211671 PMC7810473

[53] Ali N Zirak B Rodriguez RS Pauli ML Truong HA Lai K. Regulatory T cells in skin facilitate epithelial stem cell differentiation. Cell (2017) 169(6):1119–29. doi: 10.1016/j.cell.2017.05.002 PMC5504703 28552347

[54] Loser K Beissert S. Regulatory T cells: banned cells for decades. J Invest Dermatol (2012) 132(3 Pt 2):864–71. doi: 10.1038/jid.2011.375 22158548

[55] Bovenschen HJ van de Kerkhof PC van Erp PE Woestenenk R Joosten I Koenen HJPM. Foxp3+ regulatory T cells of psoriasis patients easily differentiate into IL-17A-producing cells and are found in lesional skin. J Invest Dermatol (2011) 131(9):1853–60. doi: 10.1038/jid.2011.139 21654831

[56] Pandiyan P Zhu J. Origin and functions of pro-inflammatory cytokine producing Foxp3+ regulatory T cells. Cytokine (2015) 76(1):13–24. doi: 10.1016/j.cyto.2015.07.005 26165923 PMC4969074

[57] Jung MK Kwak JE Shin EC. IL-17A-producing foxp3+ Regulatory T cells and human diseases. Immune Netw (2017) 17(5):276–86. doi: 10.4110/in.2017.17.5.276 PMC5662777 29093649

[58] Melnik BC John SM Chen W Plewig G. T helper 17 cell/regulatory T-cell imbalance in hidradenitis suppurativa/acne inversa: the link to hair follicle dissection, obesity, smoking and autoimmune comorbidities. Br J Dermatol (2018) 179(2):260–72. doi: 10.1111/bjd.16561 29573406

[59] Elias KM Laurence A Davidson TS Stephens G Kanno Y Shevach EM. Retinoic acid inhibits Th17 polarization and enhances FoxP3 expression through a Stat-3/Stat-5 independent signaling pathway. Blood (2008) 111(3):1013–20. doi: 10.1182/blood-2007-06-096438 PMC2214761 17951529

[60] Mucida D Park Y Kim G Turovskaya O Scott I Kronenberg M. Reciprocal TH17 and regulatory T cell differentiation mediated by retinoic acid. Science (2007) 317(5835):256–60. doi: 10.1126/science.1145697 17569825

[61] Dress RJ Wong AY Ginhoux F. Homeostatic control of dendritic cell numbers and differentiation. Immunol Cell Biol (2018) 96(5):463–76. doi: 10.1111/imcb.12028 29473216

[62] Guilliams M Dutertre CA Scott CL McGovern N Sichien D Chakarov S. Unsupervised high-dimensional analysis aligns dendritic cells across tissues and species. Immunity (2016) 45(3):669–84. doi: 10.1016/j.immuni.2016.08.015 PMC5040826 27637149

[63] Kashem SW Haniffa M Kaplan DH. Antigen-presenting cells in the skin. Annu Rev Immunol (2017) 35:469–99. doi: 10.1146/annurev-immunol-051116-052215 28226228

[64] Hunger RE Sieling PA Ochoa MT Sugaya M Burdick AE Rea TH. Langerhans cells utilize CD1a and langerin to efficiently present nonpeptide antigens to T cells. J Clin Invest. (2004) 113(5):701–8. doi: 10.1172/JCI200419655 PMC351318 14991068

[65] Seneschal J Clark RA Gehad A Baecher-Allan CM Kupper TS. Human epidermal Langerhans cells maintain immune homeostasis in skin by activating skin resident regulatory T cells. Immunity (2012) 36(5):873–84. doi: 10.1016/j.immuni.2012.03.018 PMC3716276 22560445

[66] Stagg AJ. Intestinal dendritic cells in health and gut inflammation. Front Immunol (2018) 9:2883. doi: 10.3389/fimmu.2018.02883 30574151 PMC6291504

[67] van Beelen AJ Zelinkova Z Taanman-Kueter EW Muller FJ Hommes DW Zaat SAJ. Stimulation of the intracellular bacterial sensor NOD2 programs dendritic cells to promote interleukin-17 production in human memory T cells. Immunity (2007) 27(4):660–9. doi: 10.1016/j.immuni.2007.08.013 17919942

[68] Farrar MD Ingham E. Acne: inflammation. Clin Dermatol (2004) 22(5):380–4. doi: 10.1016/j.clindermatol.2004.03.006 15556722

[69] Davies LC Taylor PR. Tissue-resident macrophages: then and now. Immunology (2015) 144(4):541–8. doi: 10.1111/imm.12451 PMC4368161 25684236

[70] Okabe Y Medzhitov R. Tissue-specific signals control reversible program of localization and functional polarization of macrophages. Cell (2014) 157(4):832–44. doi: 10.1016/j.cell.2014.04.016 PMC4137874 24792964

[71] Qin M Pirouz A Kim MH Krutzik SR Garbán HJ Kim J. *Propionibacterium acnes* Induces IL-1β secretion *via* the NLRP3 inflammasome in human monocytes. J Invest Dermatol (2014) 134(2):381–8. doi: 10.1038/jid.2013.309 PMC4116307 23884315

[72] Tsai HH Lee WR Wang PH Cheng KT Chen YC Shen SC. *Propionibacterium acnes*-induced iNOS and COX-2 protein expression *via* ROS-dependent NF-κB and AP-1 activation in macrophages. J Dermatol Sci (2013) 69(2):122–31. doi: 10.1016/j.jdermsci.2012.10.009 23178030

[73] Chen Q Koga T Uchi H Hara H Terao H Moroi Y. *Propionibacterium acnes*-induced IL-8 production may be mediated by NF-kappaB activation in human monocytes. J Dermatol Sci (2002) 29(2):97–103. doi: 10.1016/S0923-1811(02)00013-0 12088610

[74] Zhang B Choi YM Lee J An IS Li L He C. Toll-like receptor 2 plays a critical role in pathogenesis of acne vulgaris. BioMed Dermatol (2019) 3(1):4. doi: 10.1186/s41702-019-0042-2

[75] Fischer K Tschismarov R Pilz A Straubinger S Carotta S McDowell A. *Cutibacterium acnes* Infection Induces Type I Interferon Synthesis Through the cGAS-STING Pathway. Front Immunol (2020) 11:571334. doi: 10.3389/fimmu.2020.571334 33178195 PMC7593769

[76] Rauch I Müller M Decker T. The regulation of inflammation by interferons and their STATs. JAKSTAT (2013) 2(1):e23820. doi: 10.4161/jkst.23820 24058799 PMC3670275

[77] Zaba LC Fuentes-Duculan J Steinman RM Krueger JG Lowes MA. Normal human dermis contains distinct populations of CD11c+BDCA-1+ dendritic cells and CD163+FXIIIA+ macrophages. J Clin Invest. (2007) 117(9):2517–25. doi: 10.1172/JCI32282 PMC1957542 17786242

[78] Christoph T Müller-Röver S Audring H Tobin DJ Hermes B Cotsarelis G. The human hair follicle immune system: cellular composition and immune privilege. Br J Dermatol (2000) 142(5):862–73. doi: 10.1046/j.1365-2133.2000.03464.x 10809841

[79] Do TH Ma F Andrade PR Teles R de Andrade Silva BJ Hu C. TREM2 macrophages induced by human lipids drive inflammation in acne lesions. Sci Immunol (2022) 7(73):eabo2787. doi: 10.1126/sciimmunol.abo2787 35867799 PMC9400695

[80] Liu PT Phan J Tang D Kanchanapoomi M Hall B Krutzik SR. CD209(+) macrophages mediate host defense against *Propionibacterium acnes* . J Immunol (2008) 180(7):4919–23. doi: 10.4049/jimmunol.180.7.4919 18354216

[81] Lovászi M Mattii M Eyerich K Gácsi A Csányi E Kovács D. Sebum lipids influence macrophage polarization and activation. Br J Dermatol (2017) 177(6):1671–82. doi: 10.1111/bjd.15754 28646583

[82] Jiang H Li C. Common pathogenesis of acne vulgaris and atherosclerosis. Inflammation (2019) 42(1):1–5. doi: 10.1007/s10753-018-0863-y 30073565

[83] Pappas A Johnsen S Liu JC Eisinger M. Sebum analysis of individuals with and without acne. Dermatoendocrinol (2009) 1(3):157–61. doi: 10.4161/derm.1.3.8473 PMC2835908 20436883

[84] Kim J Lee J Kim HJ Kameyama N Nazarian R Der E. Single-cell transcriptomics applied to emigrating cells from psoriasis elucidate pathogenic versus regulatory immune cell subsets. J Allergy Clin Immunol (2021) 148(5):1281–92. doi: 10.1016/j.jaci.2021.04.021 PMC8553817 33932468

[85] Rojahn TB Vorstandlechner V Krausgruber T Bauer WM Alkon N Bangert C. Single-cell transcriptomics combined with interstitial fluid proteomics defines cell type-specific immune regulation in atopic dermatitis. J Allergy Clin Immunol (2020) 146(5):1056–69. doi: 10.1016/j.jaci.2020.03.041 32344053

[86] Weber A Knop J Maurer M. Pattern analysis of human cutaneous mast cell populations by total body surface mapping. Br J Dermatol (2003) 148(2):224–8. doi: 10.1046/j.1365-2133.2003.05090.x 12588371

[87] Valent P Akin C Hartmann K Nilsson G Reiter A Hermine O. Mast cells as a unique hematopoietic lineage and cell system: From Paul Ehrlich’s visions to precision medicine concepts. Theranostics (2020) 10(23):10743–68. doi: 10.7150/thno.46719 PMC7482799 32929378

[88] Wang Z Mascarenhas N Eckmann L Miyamoto Y Sun X Kawakami T. Skin microbiome promotes mast cell maturation by triggering stem cell factor production in keratinocytes. J Allergy Clin Immunol (2017) 139(4):1205–1216.e6. doi: 10.1016/j.jaci.2016.09.019 27746235 PMC5385284

[89] Gaudenzio N Espagnolle N Mars LT Liblau R Valitutti S Espinosa E. Cell-cell cooperation at the T helper cell/mast cell immunological synapse. Blood (2009) 114(24):4979–88. doi: 10.1182/blood-2009-02-202648 19805617

[90] Annunziato F Cosmi L Liotta F Maggi E Romagnani S. Defining the human T helper 17 cell phenotype. Trends Immunol (2012) 33(10):505–12. doi: 10.1016/j.it.2012.05.004 22682163

[91] Akamatsu H Horio T. The possible role of reactive oxygen species generated by neutrophils in mediating acne inflammation. Dermatology (1998) 196(1):82–5. doi: 10.1159/000017876 9557235

[92] Nakai K Tsuruta D. What are reactive oxygen species, free radicals, and oxidative stress in skin diseases? Int J Mol Sci (2021) 22(19):10799. doi: 10.3390/ijms221910799 34639139 PMC8509443

[93] Beringer A Noack M Miossec P. IL-17 in chronic inflammation: from discovery to targeting. Trends Mol Med (2016) 22(3):230–41. doi: 10.1016/j.molmed.2016.01.001 26837266

[94] Cua DJ Tato CM. Innate IL-17-producing cells: the sentinels of the immune system. Nat Rev Immunol (2010) 10(7):479–89. doi: 10.1038/nri2800 20559326

[95] Hazenberg MD Spits H. Human innate lymphoid cells. Blood (2014) 124(5):700–9. doi: 10.1182/blood-2013-11-427781 24778151

[96] Ghaedi M Takei F. Innate lymphoid cell development. J Allergy Clin Immunol (2021) 147(5):1549–60. doi: 10.1016/j.jaci.2021.03.009 33965092

[97] Kobayashi T Voisin B Kim DY Kennedy EA Jo JH Shih HY. Homeostatic control of sebaceous glands by innate lymphoid cells regulates commensal bacteria equilibrium. Cell (2019) 176(5):982–997.e16. doi: 10.1016/j.cell.2018.12.031 30712873 PMC6532063

[98] Petrasca A Hambly R Molloy O Kearns S Moran B Smith CM. Innate lymphoid cell (ILC) subsets are enriched in the skin of patients with hidradenitis suppurativa. PloS One (2023) 18(2):e0281688. doi: 10.1371/journal.pone.0281688 36780439 PMC9924995

[99] Teunissen MBM Munneke JM Bernink JH Spuls PI Res PCM Te Velde A. Composition of innate lymphoid cell subsets in the human skin: enrichment of NCR(+) ILC3 in lesional skin and blood of psoriasis patients. J Invest Dermatol (2014) 134(9):2351–60. doi: 10.1038/jid.2014.146 24658504

[100] Montaldo E Juelke K Romagnani C. Group 3 innate lymphoid cells (ILC3s): Origin, differentiation, and plasticity in humans and mice. Eur J Immunol (2015) 45(8):2171–82. doi: 10.1002/eji.201545598 26031799

[101] Nestle FO Di Meglio P Qin JZ Nickoloff BJ. Skin immune sentinels in health and disease. Nat Rev Immunol (2009) 9(10):679–91. doi: 10.1038/nri2622 PMC2947825 19763149

[102] Jiang Y Tsoi LC Billi AC Ward NL Harms PW Zeng C. Cytokinocytes: the diverse contribution of keratinocytes to immune responses in skin. JCI Insight (2020) 5(20):142067. doi: 10.1172/jci.insight.142067 33055429 PMC7605526

[103] Kupper TS Ballard DW Chua AO McGuire JS Flood PM Horowitz MC. Human keratinocytes contain mRNA indistinguishable from monocyte interleukin 1 alpha and beta mRNA. Keratinocyte epidermal cell-derived thymocyte-activating factor is identical to interleukin 1. J Exp Med (1986) 164(6):2095–100. doi: 10.1084/jem.164.6.2095 PMC2188493 2431094

[104] Nagy I Pivarcsi A Koreck A Széll M Urbán E Kemény L. Distinct strains of *Propionibacterium acnes* induce selective human beta-defensin-2 and interleukin-8 expression in human keratinocytes through toll-like receptors. J Invest Dermatol (2005) 124(5):931–8. doi: 10.1111/j.0022-202X.2005.23705.x 15854033

[105] Jugeau S Tenaud I Knol AC Jarrousse V Quereux G Khammari A. Induction of toll-like receptors by *Propionibacterium acnes* . Br J Dermatol (2005) 153(6):1105–13. doi: 10.1111/j.1365-2133.2005.06933.x 16307644

[106] Graham GM Farrar MD Cruse-Sawyer JE Holland KT Ingham E. Proinflammatory cytokine production by human keratinocytes stimulated with *Propionibacterium acnes* and *P.* acnes GroEL. Br J Dermatol (2004) 150(3):421–8. doi: 10.1046/j.1365-2133.2004.05762.x 15030323

[107] Schaller M Loewenstein M Borelli C Jacob K Vogeser M Burgdorf WHC. Induction of a chemoattractive proinflammatory cytokine response after stimulation of keratinocytes with *Propionibacterium acnes* and coproporphyrin III. Br J Dermatol (2005) 153(1):66–71. doi: 10.1111/j.1365-2133.2005.06530.x 16029328

[108] Grange PA Chéreau C Raingeaud J Nicco C Weill B Dupin N. Production of superoxide anions by keratinocytes initiates *P. acnes*-induced inflammation of the skin. PloS Pathog (2009) 5(7):e1000527. doi: 10.1371/journal.ppat.1000527 19629174 PMC2709429

[109] Ottaviani M Alestas T Flori E MastroFrancesco A Zouboulis CC Picardo M. Peroxidated squalene induces the production of inflammatory mediators in HaCaT keratinocytes: a possible role in acne vulgaris. J Invest Dermatol (2006) 126(11):2430–7. doi: 10.1038/sj.jid.5700434 16778793

[110] Leire E Olson J Isaacs H Nizet V Hollands A. Role of hypoxia inducible factor-1 in keratinocyte inflammatory response and neutrophil recruitment. J Inflammation (Lond). (2013) 10(1):28. doi: 10.1186/1476-9255-10-28 PMC3751314 23937964

[111] Zouboulis CC Yoshida GJ Wu Y Xia L Schneider MR. Sebaceous gland: Milestones of 30-year modelling research dedicated to the “brain of the skin”. Exp Dermatol (2020) 29(11):1069–79. doi: 10.1111/exd.14184 32875660

[112] Schneider MR Schmidt-Ullrich R Paus R. The hair follicle as a dynamic miniorgan. Curr Biol (2009) 19(3):R132–142. doi: 10.1016/j.cub.2008.12.005 19211055

[113] Fischer H Fumicz J Rossiter H Napirei M Buchberger M Tschachler E. Holocrine secretion of sebum is a unique DNase2-dependent mode of programmed cell death. J Invest Dermatol (2017) 137(3):587–94. doi: 10.1016/j.jid.2016.10.017 27771328

[114] Schneider MR Paus R. Sebocytes, multifaceted epithelial cells: lipid production and holocrine secretion. Int J Biochem Cell Biol (2010) 42(2):181–5. doi: 10.1016/j.biocel.2009.11.017 19944183

[115] Camera E Ludovici M Galante M Sinagra JL Picardo M. Comprehensive analysis of the major lipid classes in sebum by rapid resolution high-performance liquid chromatography and electrospray mass spectrometry. J Lipid Res (2010) 51(11):3377–88. doi: 10.1194/jlr.D008391 PMC2952580 20719760

[116] Smith KR Thiboutot DM. Thematic review series: skin lipids. Sebaceous gland lipids: friend or foe? J Lipid Res (2008) 49(2):271–81. doi: 10.1194/jlr.R700015-JLR200 17975220

[117] Zouboulis CC Picardo M Ju Q Kurokawa I Törőcsik D Bíró T. Beyond acne: Current aspects of sebaceous gland biology and function. Rev Endocr Metab Disord (2016) 17(3):319–34. doi: 10.1007/s11154-016-9389-5 27726049

[118] Kovács D Lovászi M Póliska S Oláh A Bíró T Veres I. Sebocytes differentially express and secrete adipokines. Exp Dermatol (2016) 25(3):194–9. doi: 10.1111/exd.12879 26476096

[119] Rao A Douglas SC Hall JM. Endocrine disrupting chemicals, hormone receptors, and acne vulgaris: A connecting hypothesis. Cells (2021) 10(6):1439. doi: 10.3390/cells10061439 34207527 PMC8228950

[120] Cappel M Mauger D Thiboutot D. Correlation between serum levels of insulin-like growth factor 1, dehydroepiandrosterone sulfate, and dihydrotestosterone and acne lesion counts in adult women. Arch Dermatol (2005) 141(3):333–8. doi: 10.1001/archderm.141.3.333 15781674

[121] Kim H Moon SY Sohn MY Lee WJ. Insulin-like growth factor-1 increases the expression of inflammatory biomarkers and sebum production in cultured sebocytes. Ann Dermatol (2017) 29(1):20–5. doi: 10.5021/ad.2017.29.1.20 PMC5318522 28223742

[122] Georgel P Crozat K Lauth X Makrantonaki E Seltmann H Sovath S. A toll-like receptor 2-responsive lipid effector pathway protects mammals against skin infections with gram-positive bacteria. Infect Immun (2005) 73(8):4512–21. doi: 10.1128/IAI.73.8.4512-4521.2005 PMC1201198 16040962

[123] Nagy I Pivarcsi A Kis K Koreck A Bodai L McDowell A. *Propionibacterium acnes* and lipopolysaccharide induce the expression of antimicrobial peptides and proinflammatory cytokines/chemokines in human sebocytes. Microbes Infect (2006) 8(8):2195–205. doi: 10.1016/j.micinf.2006.04.001 16797202

[124] Li ZJ Choi DK Sohn KC Seo MS Lee HE Lee Y. *Propionibacterium acnes* activates the NLRP3 inflammasome in human sebocytes. J Invest Dermatol (2014) 134(11):2747–56. doi: 10.1038/jid.2014.221 24820890

[125] Huang YC Yang CH Li TT Zouboulis CC Hsu HC. Cell-free extracts of *Propionibacterium acnes* stimulate cytokine production through activation of p38 MAPK and Toll-like receptor in SZ95 sebocytes. Life Sci (2015) 139:123–31. doi: 10.1016/j.lfs.2015.07.028 26341693

[126] Oulès B Philippeos C Segal J Tihy M Vietri Rudan M Cujba AM. Contribution of GATA6 to homeostasis of the human upper pilosebaceous unit and acne pathogenesis. Nat Commun (2020) 11(1):5067. doi: 10.1038/s41467-020-18784-z 33082341 PMC7575575

[127] Stelzner K Herbert D Popkova Y Lorz A Schiller J Gericke M. Free fatty acids sensitize dendritic cells to amplify TH1/TH17-immune responses. Eur J Immunol (2016) 46(8):2043–53. doi: 10.1002/eji.201546263 27214608

[128] Choi CW Kim Y Kim JE Seo EY Zouboulis CC Kang JS. Enhancement of lipid content and inflammatory cytokine secretion in SZ95 sebocytes by palmitic acid suggests a potential link between free fatty acids and acne aggravation. Exp Dermatol (2019) 28(2):207–10. doi: 10.1111/exd.13855 30506807

[129] Lee SE Kim JM Jeong SK Choi EH Zouboulis CC Lee SH. Expression of protease-activated receptor-2 in SZ95 sebocytes and its role in sebaceous lipogenesis, inflammation, and innate immunity. J Invest Dermatol (2015) 135(9):2219–27. doi: 10.1038/jid.2015.151 25880702

[130] Nakatsuji T Kao MC Zhang L Zouboulis CC Gallo RL Huang CM. Sebum free fatty acids enhance the innate immune defense of human sebocytes by upregulating beta-defensin-2 expression. J Invest Dermatol (2010) 130(4):985–94. doi: 10.1038/jid.2009.384 PMC3057125 20032992

[131] Sanford JA Zhang LJ Williams MR Gangoiti JA Huang CM Gallo RL. Inhibition of HDAC8 and HDAC9 by microbial short-chain fatty acids breaks immune tolerance of the epidermis to TLR ligands. Sci Immunol (2016) 1(4):eaah4609. doi: 10.1126/sciimmunol.aah4609 28783689

[132] Sanford JA O’Neill AM Zouboulis CC Gallo RL. Short-chain fatty acids from *cutibacterium acnes* activate both a canonical and epigenetic inflammatory response in human sebocytes. J Immunol (2019) 202(6):1767–76. doi: 10.4049/jimmunol.1800893 PMC7251550 30737272

[133] Li X He C Chen Z Zhou C Gan Y Jia Y. A review of the role of sebum in the mechanism of acne pathogenesis. J Cosmet Dermatol (2017) 16(2):168–73. doi: 10.1111/jocd.12345 28556292

[134] Camera E Ludovici M Tortorella S Sinagra JL Capitanio B Goracci L. Use of lipidomics to investigate sebum dysfunction in juvenile acne. J Lipid Res (2016) 57(6):1051–8. doi: 10.1194/jlr.M067942 PMC4878189 27127078

[135] Tochio T Tanaka H Nakata S Ikeno H. Accumulation of lipid peroxide in the content of comedones may be involved in the progression of comedogenesis and inflammatory changes in comedones. J Cosmet Dermatol (2009) 8(2):152–8. doi: 10.1111/j.1473-2165.2009.00437.x 19527342

[136] Zouboulis CC Angres S Seltmann H. Regulation of stearoyl-coenzyme A desaturase and fatty acid delta-6 desaturase-2 expression by linoleic acid and arachidonic acid in human sebocytes leads to enhancement of proinflammatory activity but does not affect lipogenesis. Br J Dermatol (2011) 165(2):269–76. doi: 10.1111/j.1365-2133.2011.10340.x 21457203

[137] Capitanio B Lora V Ludovici M Sinagra JL Ottaviani M MastroFrancesco A. Modulation of sebum oxidation and interleukin-1α levels associates with clinical improvement of mild comedonal acne. J Eur Acad Dermatol Venereol. (2014) 28(12):1792–7. doi: 10.1111/jdv.12431 24628899

[138] Snodgrass RG Huang S Choi IW Rutledge JC Hwang DH. Inflammasome-mediated secretion of IL-1β in human monocytes through TLR2 activation; modulation by dietary fatty acids. J Immunol (2013) 191(8):4337–47. doi: 10.4049/jimmunol.1300298 PMC3825708 24043885

[139] Törőcsik D Fazekas F Póliska S Gregus A Janka EA Dull K. Epidermal growth factor modulates palmitic acid-induced inflammatory and lipid signaling pathways in SZ95 sebocytes. Front Immunol (2021) 12:600017. doi: 10.3389/fimmu.2021.600017 34025636 PMC8134683

[140] Maya-Monteiro CM Bozza PT. Leptin and mTOR: partners in metabolism and inflammation. Cell Cycle (2008) 7(12):1713–7. doi: 10.4161/cc.7.12.6157 18583936

[141] Conde J Scotece M Abella V López V Pino J Gómez-Reino JJ. An update on leptin as immunomodulator. Expert Rev Clin Immunol (2014) 10(9):1165–70. doi: 10.1586/1744666X.2014.942289 25098336

[142] Törőcsik D Kovács D Camera E Lovászi M Cseri K Nagy GG. Leptin promotes a proinflammatory lipid profile and induces inflammatory pathways in human SZ95 sebocytes. Br J Dermatol (2014) 171(6):1326–35. doi: 10.1111/bjd.13229 24975960

[143] Rees BS Lee K Fanok MH Mascaraque C Amoury M Chon LB. Leptin receptor signaling in T cells is required for Th17 differentiation. J Immunol (Baltimore Md: 1950) (2015) 194(11):5253–60. doi: 10.4049/jimmunol.1402996 PMC4433844 25917102

[144] Melnik BC. Is sebocyte-derived leptin the missing link between hyperseborrhea, ductal hypoxia, inflammation and comedogenesis in acne vulgaris? Exp Dermatol (2016) 25(3):181–2. doi: 10.1111/exd.12917 26660941

[145] Chen SX Zhang LJ Gallo RL. Dermal white adipose tissue: A newly recognized layer of skin innate defense. J Invest Dermatol (2019) 139(5):1002–9. doi: 10.1016/j.jid.2018.12.031 30879642

[146] Zhang L-j Guerrero-Juarez CF Hata T Bapat SP Ramos R Plikus MV. Dermal adipocytes protect against invasive *Staphylococcus aureus* skin infection. Science (2015) 347(6217):67–71. doi: 10.1126/science.1260972 25554785 PMC4318537

[147] O’Neill AM Liggins MC Seidman JS Do TH Li F Cavagnero KJ. Antimicrobial production by perifollicular dermal preadipocytes is essential to the pathophysiology of acne. Sci Transl Med (2022) 14(632):eabh1478. doi: 10.1126/scitranslmed.abh1478 35171653 PMC9885891

